# An ontological foundation for ocular phenotypes and rare eye diseases

**DOI:** 10.1186/s13023-018-0980-6

**Published:** 2019-01-09

**Authors:** Panagiotis I. Sergouniotis, Emmanuel Maxime, Dorothée Leroux, Annie Olry, Rachel Thompson, Ana Rath, Peter N. Robinson, Hélène Dollfus, for the ERN-EYE Ontology Study Group, Jane L. Ashworth, Isabelle Audo, Vilma Jurate Balciuniene, Eyal Banin, Graeme C. Black, Daniel Böhringer, Camiel J. F. Boon, Dominique Bremond-Gignac, Patrick Calvas, Guilherme Castela, Gislin Dagnelie, Hélène Dollfus, Susan M. Downes, Adriano Fasolo, Christina Fasser, Arvydas Gelzinis, Kerry Goetz, Steffen Hamann, Elise Héon, Giancarlo Iarossi, Aki Kawasaki, David Keegan, Line Kessel, Kamron Khan, Artur Klett, Sebastian Köhler, Dorothée Leroux, Bart P. Leroy, Walter Lisch, Petra Liskova, Birgit Lorenz, Riccardo Maggi, Emmanuel Maxime, Isabelle Meunier, Saddek Mohand-Said, Katarzyna Nowomiejska, Yaumara Perdomo, Axel Petzold, Markus Preising, Peter N. Robinson, Hendrik P. N. Scholl, Panagiotis I. Sergouniotis, Andrea Sodi, Katarina Stingl, Fouzia Studer, Agnese Suppiej, Rachel Thompson, Valerie Touitou, Elias Traboulsi, Jurgis Trumpaitis, Stephen J. Tuft, Veronika Vaclavik, Sandra Valeina, Caroline Van Cauwenbergh, Alain Verloes, Alain Vighetto, Russell Wheeler, Thomas Wheeler-Schilling, Patrick Yu-Wai-Man, Ditta Zobor, Eberhart Zrenner

**Affiliations:** 1 University of Manchester and Manchester Royal Eye Hospital, Oxford Road, Manchester, M13 9WL UK; 2 Orphanet, INSERM (Institut National de la Santé et de la Recherche Médicale), Paris, France; 3 Centre for Rare Eye Diseases CARGO, SENSGENE FSMR Network, Strasbourg University Hospital, Strasbourg, France; 4 Newcastle University, Newcastle upon Tyne, UK; 5 The Jackson Laboratory for Genomic Medicine, Farmington, CT USA; 6 Laboratoire de Génétique Médicale, Faculté de Médecine de Strasbourg, INSERM U1112, 11 rue Humann, 67 085 Strasbourg, France

**Keywords:** Evidence-based precision medicine, Rare eye disease, Human phenotype ontology, Orphanet rare disease ontology

## Abstract

**Background:**

The optical accessibility of the eye and technological advances in ophthalmic diagnostics have put ophthalmology at the forefront of data-driven medicine. The focus of this study is rare eye disorders, a group of conditions whose clinical heterogeneity and geographic dispersion make data-driven, evidence-based practice particularly challenging. Inter-institutional collaboration and information sharing is crucial but the lack of standardised terminology poses an important barrier. Ontologies are computational tools that include sets of vocabulary terms arranged in hierarchical structures. They can be used to provide robust terminology standards and to enhance data interoperability. Here, we discuss the development of the ophthalmology-related component of two well-established biomedical ontologies, the Human Phenotype Ontology (HPO; includes signs, symptoms and investigation findings) and the Orphanet Rare Disease Ontology (ORDO; includes rare disease nomenclature/nosology).

**Methods:**

A variety of approaches were used including automated matching to existing resources and extensive manual curation. To achieve the latter, a study group including clinicians, patient representatives and ontology developers from 17 countries was formed. A broad range of terms was discussed and validated during a dedicated workshop attended by 60 members of the group.

**Results:**

A comprehensive, structured and well-defined set of terms has been agreed on including 1106 terms relating to ocular phenotypes (HPO) and 1202 terms relating to rare eye disease nomenclature (ORDO). These terms and their relevant annotations can be accessed in http://www.human-phenotype-ontology.org/ and http://www.orpha.net/; comments, corrections, suggestions and requests for new terms can be made through these websites. This is an ongoing, community-driven endeavour and both HPO and ORDO are regularly updated.

**Conclusions:**

To our knowledge, this is the first effort of such scale to provide terminology standards for the rare eye disease community. We hope that this work will not only improve coding and standardise information exchange in clinical care and research, but also it will catalyse the transition to an evidence-based precision ophthalmology paradigm.

**Electronic supplementary material:**

The online version of this article (10.1186/s13023-018-0980-6) contains supplementary material, which is available to authorized users.

Rare eye diseases (REDs) are a major cause of visual impairment and blindness in children and young adults [1, 2]. To date, according to Orphanet, over 1000 REDs have been described; the majority of these conditions are genetic and many of them have prominent extraocular features [3]. Although each individual disorder is rare (defined in the European Union as affecting less than 1 in 2000 individuals [4]), collectively they are common and their cumulative impact on affected families and healthcare systems is substantial [5, 6].

The rarity of each RED, together with the significant clinical and genetic heterogeneity that characterises this group of conditions, can make precise diagnosis and evidence-based management challenging. To improve patient care and to obtain sufficient sample sizes for research, responsible sharing of knowledge and data across centres and countries is required [7]. Adoption of comprehensive phenotype and rare disease ontologies enables this type of sharing by making data findable, accessible, interoperable, and re-usable (FAIR principles) [8].

Ontologies are computational tools assisting in the description, organisation and analysis of data. An ontology provides not only a standardised set of vocabulary terms but also a classification of these entities so that terms with related meanings are connected by well-defined relationships. Central to each ontology are terms, also known as classes, which are arranged in a hierarchical and semantically informative structure from the general (high in the hierarchy) to the specific (low in the hierarchy) [9–11] (Fig. 1; Additional file 1: Figure S1).

**Fig. 1:**
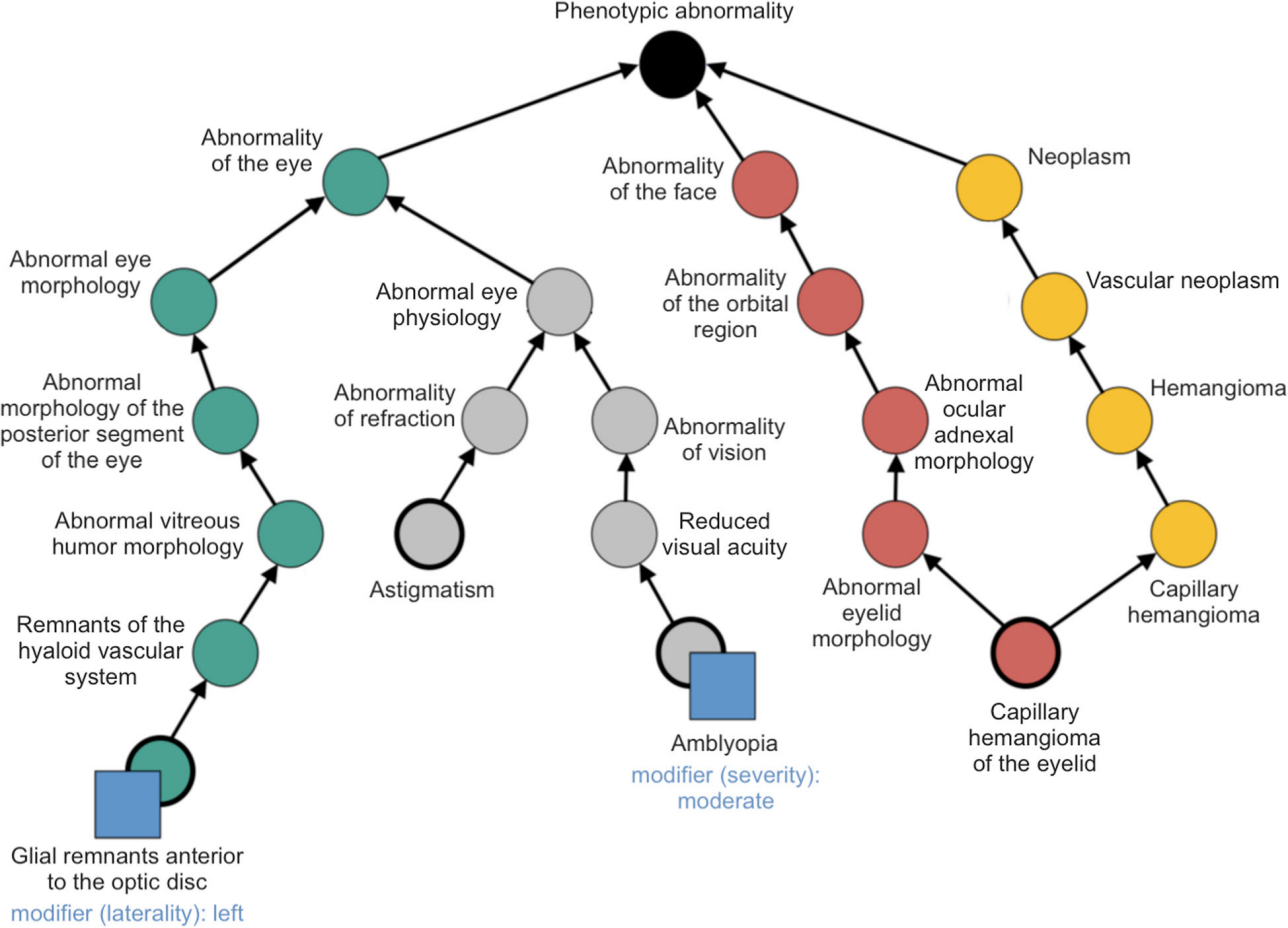
Example of hierarchical (tree) structure of data in the Human Phenotype Ontology (HPO). Ophthalmic findings in a child with PHACE syndrome (posterior fossa anomalies, hemangioma, arterial lesions, cardiac abnormalities/coarctation of the aorta, eye anomalies) are shown. Ontologies consist of several distinct elements including terms (nodes in the figure) and relationships (arrows in the figure). Each term can be associated with annotated textual information known as metadata; these may include modifiers (blue squares), definitions and alternative/secondary identifiers. Modifiers can be assigned to each term and may relate to severity (mild, moderate etc.), temporal pattern (acute, episodic, etc.), age of onset (childhood onset, adult onset etc), progression (progressive, nonprogressive, etc.), laterality (right, bilateral etc) and spatial pattern (central, generalized, etc.). The user can specify if a specific phenotype (HPO term) is present or absent in an individual. When a phenotype/term is selected as present (e.g. terms corresponding to the five circles with thickened margins) then, by definition, all terms above this term (coloured circles; colours only used to enhance visualisation) have to be present; this is because each term is connected with its parent terms by an “is a” relationship. Therefore, the higher in the ontology a term is located, the more general it is and the lesser its information content (defined as the negative logarithm of its probability) will be

This work focuses on developing the ophthalmology-related component of two widely utilized biomedical ontologies, the Human Phenotype Ontology (HPO) and the Orphanet Rare Disease Ontology (ORDO) [3, 10, 12, 13]. Each term in HPO describes a distinct phenotypic feature such as a symptom (e.g. photophobia) or a sign (e.g. Haab’s striae), while each ORDO term corresponds to a specific rare disorder (e.g. posterior polymorphous corneal dystrophy).

To set a robust ontological foundation for ocular phenotypes and REDs a multi-step approach was utilized. The HPO project commenced in 2007 with the initial aim of aiding rare disease phenotyping and diagnostics [9]. Data from the Online Mendelian Inheritance in Man (OMIM; https://omim.org/) database were used for the construction of HPO and, over the past decade, significant refinement and expansion occurred through manual curation and careful mapping to other resources including the Systematized Nomenclature of Medicine-Clinical Terms (SNOMED-CT; https://www.snomed.org/snomed-ct) and the International Statistical Classification of Diseases and Related Health Problems (ICD; http://www.who.int/classifications/icd/). The latest release of HPO (11/2018) includes 1106 terms related to ocular phenotypes. There are 842 textual and/or logical definitions and 968 synonyms related to these terms; 1208 subclass relationships and 7702 annotations of ocular phenotypes to 2770 rare disorders have been created. A set of tools that can be used to explore HPO can be found in https://hpo.jax.org/app/tools/hpo-browser and https://hpo.jax.org/app/tools/workbench.

Orphanet is an international data resource that was created in 1997 to address the scarcity and fragmentation of information on rare diseases. This unique database has grown substantially over the past 20 years by incorporating expert advice and through extensive manual curation of medical literature. Importantly, there is almost complete linkage of Orphanet with SNOMED-CT and ICD, two resources that are commonly used in electronic healthcare records (EHRs) but are presently less rich in rare disease related content. The Orphanet nomenclature evolved into a formal ontology (ORDO) in 2014 and the latest version (11/2018) includes 1202 RED-related entries (including 146 groups, 834 disorders and 222 subtypes) each having a unique, stable ORPHA number. The Orphanet nomenclature can be browsed in https://www.orpha.net and ORDO is available at http://www.orphadata.org/cgi-bin/index.php#ontologies.

Our efforts to enrich the ophthalmology-related content of these two resources culminated in a 4-day workshop in October 2017 (ERN-EYE Ontology meeting, Mont Sainte-Odile, France). In this meeting, 60 members of the European Reference Network on rare eye diseases (ERN-EYE) from 29 centres of 13 countries [14] worked with international experts to enrich the HPO and Orphanet classifications and to agree on a broad range of terms. Briefly, the adopted approach involved sharing existing HPO terms and Orphanet classifications with all ERN-EYE members prior to the workshop. Participants were distributed in six working groups (retina, neuro-ophthalmology, paediatric ophthalmology, anterior segment, low vision, and genetic diagnostics) who discussed issues and suggestions. Patient representatives were also consulted and their active participation at the workshop provided key insights. Subsequent engagement of ERN-EYE members has led to further refinement of the ontologies including providing definitions and other relevant annotations. Overall, 605 new HPO terms related to ocular disease were added and over 400 terms were revised (including adding definitions and/or synonyms, correcting errors and removing/merging obsolete terms). The Orphanet classification was fully restructured with more than 67 groups created, 36 disorders introduced, 90 entities removed/merged, and 131 modifications made in the nomenclature. For further information on the specific changes made as a result of the workshop please see Additional file 1: Tables S1 and S2.

There are multiple advantages in using ontologies, the most important one being enhanced data integration: by making data computationally accessible it is possible to bridge the compatibility gap between different healthcare systems, databases and languages. Also, sophisticated cross-resource bioinformatics analyses with data from other domains (e.g. genomic data) is enabled. These features have made ORPHA numbers a standard for rare disease coding in European healthcare systems and have led to the widespread adoption of ontologies like HPO by global genomics initiatives (e.g. Matchmaker Exchange [15] and RD-Connect [16]) and clinical genetic laboratories. Notably, both ORDO and HPO are open-access, interoperable, community-driven, available in multiple languages and regularly updated (twice a year for ORDO, at least quarterly for HPO). Requests for new terms or other amendments can be made through the Orphanet and HPO portals and we hope to engage all stakeholders in this ongoing effort. The ERN-EYE remains committed to further improving these resources and future plans include working on “layperson” terms for ocular phenotypes (so that patients can also use HPO) and on annotating each RED with HPO terms (to enhance diagnostic accuracy) [17].

To our knowledge, this is the first effort of such scale to provide terminology standards for the RED community. The resources outlined in this article have the potential to transform both routine care (e.g. by integration in EHRs) and research (e.g. recording clinical data in registries) for RED. We believe that collaborative efforts like this not only result in improved data classification and coding, but also lead to growing acceptance of standard nomenclatures among the RED community. Finally, we hope that this work will catalyse responsible inter-institutional data sharing and facilitate the transition to an evidence-based precision medicine paradigm for RED [18].

## Additional file

Additional file 1. Example of hierarchical data structure in ORDO (**Figure S1**), summary of the modifications made to HPO and ORDO as part of this study (**Table S1**), and list of disease groups and specific disorders introduced to ORDO as a result of the ERN-EYE Ontology meeting (Mont Sainte-Odile, France, 10/2017) (**Table S2**).ᅟ(PDF 428 kb)

## Abbreviations

EHR: Electronic healthcare record
ERN-EYE: European Reference Network on rare eye diseases
HPO: Human Phenotype Ontology
ICD: International Statistical Classification of Diseases and Related Health Problems
OMIM: Online Mendelian Inheritance in Man
ORDO: Orphanet Rare Disease Ontology
RED: rare eye disease
SNOMED-CT: Systematized Nomenclature of Medicine-Clinical Terms
