# Correction to: An ontological foundation for ocular phenotypes and rare eye diseases

**DOI:** 10.1186/s13023-019-1156-8

**Published:** 2019-08-15

**Authors:** Panagiotis I. Sergouniotis, Emmanuel Maxime, Dorothée Leroux, Annie Olry, Rachel Thompson, Ana Rath, Peter N. Robinson, Hélène Dollfus, for the ERN-EYE Ontology Study Group

**Affiliations:** 1 University of Manchester and Manchester Royal Eye Hospital, Oxford Road, Manchester, M13 9WL UK; 2 Orphanet, INSERM (Institut National de la Santé et de la Recherche Médicale), Paris, France; 3 Centre for Rare Eye Diseases CARGO, SENSGENE FSMR Network, Strasbourg University Hospital, Strasbourg, France; 4 Newcastle University, Newcastle upon Tyne, UK; 5 The Jackson Laboratory for Genomic Medicine, Farmington, CT USA; 6 Laboratoire de Génétique Médicale, Faculté de Médecine de Strasbourg, INSERM U1112, 11 rue Humann, 67 085 Strasbourg, France

**Correction to: Orphanet J Rare Dis**

**https://doi.org/10.1186/s13023-018-0980-6**

Professor Michael Larsen, who is a member of the ERN-EYE Ontology Study Group and co-chair of Workgroup on Retinal Rare Eye Diseases (WG1), was inadvertently omitted from the author list in the Acknowledgements section of the original article [1].
