# Supplementary material for: An ontological foundation for ocular phenotypes and rare eye diseases
Source: Orphanet J Rare Dis. 2019 Jan 9;14:8. doi: 10.1186/s13023-018-0980-6 (PMC6327432; doi:10.1186/s13023-018-0980-6)
Supplement: Supplementary file 1 — Example of hierarchical data structure in ORDO (Figure S1), summary of the modifications made to HPO and ORDO as part of this study (Table S1), and list of disease groups and specific disorders introduced to ORDO as a result of the ERN-EYE Ontology meeting (Mont Sainte-Odile, France, 10/2017) (Table S2).ᅟ(PDF 428 kb) [file 13023_2018_980_MOESM1_ESM.pdf]

Additional File

An ontological foundation for ocular phenotypes and rare eye diseases

Panagiotis I. Sergouniotis[1], Emmanuel Maxime[2], Dorothée Leroux[3], Annie Olry[2], Rachel Thompson[4], Ana Rath[2], Peter N. Robinson[5], and Hélène Dollfus[3] on behalf of the ERN-EYE Ontology Study Group†

[1] University of Manchester & Manchester Royal Eye Hospital, Manchester, UK.

[2] Orphanet, INSERM (Institut National de la Santé et de la Recherche Médicale), Paris, France.

[3] Centre for Rare Eye Diseases CARGO, SENSGENE FSMR Network, Strasbourg University Hospital, Strasbourg, France.

[4] Newcastle University, Newcastle upon Tyne, UK.

[5] The Jackson Laboratory for Genomic Medicine, Farmington, US.

† ERN-EYE Ontology Study Group:

Jane L. Ashworth[1], Isabelle Audo[6], Vilma Jurate Balciuniene[7], Eyal Banin[8], Graeme C. Black[1], Daniel Böhringer[9], Camiel J.F. Boon[10,11], Dominique Bremond-Gignac[12], Patrick Calvas[13], Guilherme Castela[14], Gislin Dagnelie[15], Susan M. Downes[16], Adriano Fasolo[17], Christina Fasser[18], Arvydas Gelzinis[7], Kerry Goetz[19], Steffen Hamann[20], Elise Héon[21], Giancarlo Iarossi[22], Aki Kawasaki[23], David Keegan[24], Line Kessel[20], Kamron Khan[25], Artur Klett[26], Sebastian Köhler[27], Bart P. Leroy[28], Walter Lisch[29], Petra Liskova[30], Birgit Lorenz[31], Riccardo Maggi[22], Isabelle Meunier[32], Saddek Mohand-Said[6], Katarzyna Nowomiejska[33], Yaumara Perdomo[3], Axel Petzold[34], Markus Preising[31], Hendrik P.N. Scholl[35,36,37], Andrea Sodi[38], Katarina Stingl[39], Fouzia Studer[3], Agnese Suppiej[40], Valerie Touitou[41], Elias Traboulsi[42], Jurgis Trumpaitis[7], Stephen J. Tuft[34], Veronika Vaclavik[43], Sandra Valeina[44], Caroline Van Cauwenbergh[28], Alain Verloes[45], Alain Vighetto[46], Russell Wheeler[47], Thomas Wheeler-Schilling[39], Patrick Yu-Wai-Man[34,48], Ditta Zobor[39], Eberhart Zrenner[39].

[6] Quinze-Vingts National Ophthalmology Hospital, Paris, France.

[7] Hospital of Lithuanian University of Health Science, Kaunas, Lithuania.

[8] Hadassah Medical Center, Jerusalem, Israel.

[9] Eye Centre, University Hospital Freiburg, Germany.

[10] Academic Medical Centre, University of Amsterdam, Amsterdam, the Netherlands.

[11] Leiden University Medical Center, Leiden, the Netherlands.

[12] University Hospital Necker-Enfants Malades, Paris, France.

[13] University of Toulouse, Toulouse, France.

[14] University of Coimbra, Coimbra, Portugal.

[15] Johns Hopkins University School of Medicine, Baltimore, USA.

[16] Nuffield Department of Clinical Neuroscience, John Radcliffe Hospital, Oxford, UK.

[17] The Veneto Eye Bank Foundation, Venice, Italy.

[18] Retina International, Zürich, Switzerland.

[19] National Eye Institute, Bethesda, USA.

[20] Department of Ophthalmology, Rigshospitalet, University of Copenhagen, Glostrup, Denmark.

[21] The Hospital for Sick Children, Toronto, Canada.

[22] Bambino Gesù Children's Hospital, Rome, Italy.

[23] University of Lausanne, Lausanne, Switzerland.

[24] Mater Misericordiae University Hospital, Dublin, Ireland.

[25] St James's University Hospital, Leeds, UK.

[26] Eye Clinic of East Tallinn Central Hospital, Tallinn, Estonia

[27] Charité-Universitätsmedizin Berlin, Berlin, Germany.

[28] Ghent University & Ghent University Hospital, Ghent, Belgium.

[29] Johannes Gutenberg University Mainz, Mainz, Germany.

[30] Charles University & General University Hospital in Prague, Prague, Czech Republic.

[31] Justus Liebig University, Giessen, Germany.

[32] University of Montpellier, Montpellier, France.

[33] Medical University of Lublin, Lublin, Poland.

[34] Moorfields Eye Hospital, London, UK.

[35] Institute of Molecular & Clinical Ophthalmology Basel, Basel, Switzerland.

[36] Department of Ophthalmology, University of Basel, Basel, Switzerland.

[37] Wilmer Eye Institute, Johns Hopkins University, Baltimore, United States.

[38] Careggi Teaching Hospital, Florence, Italy.

[39] University of Tübingen, Tübingen, Germany.

[40] Pediatric University Hospital of Padova, Italy.

[41] Pitie-Salpetriere Hospital, Paris, France.

[42] Cleveland Clinic, Cleveland, USA.

[43] Jules-Gonin Eye Hospital, Lausanne, Switzerland.

[44] Children's Clinical University Hospital, Riga, Latvia.

[45] APHP - University Hospital Robert Debré, Paris, France.

[46] Hospital for Neurology & Neurosurgery Pierre Wertheimer, Bron, France.

[47] Leber's Hereditary Optic Neuropathy (LHON) Society, UK.

[48] University of Cambridge, Cambridge, UK.

Figure S1

Figure S1: Example of hierarchical (tree) structure of data in the Orphanet Rare Disease Ontology (ORDO). PHACE syndrome (posterior fossa anomalies, hemangioma, arterial lesions, cardiac abnormalities/coarctation of the aorta, eye anomalies) is used as an example; only the ocular component of the disorder is shown.

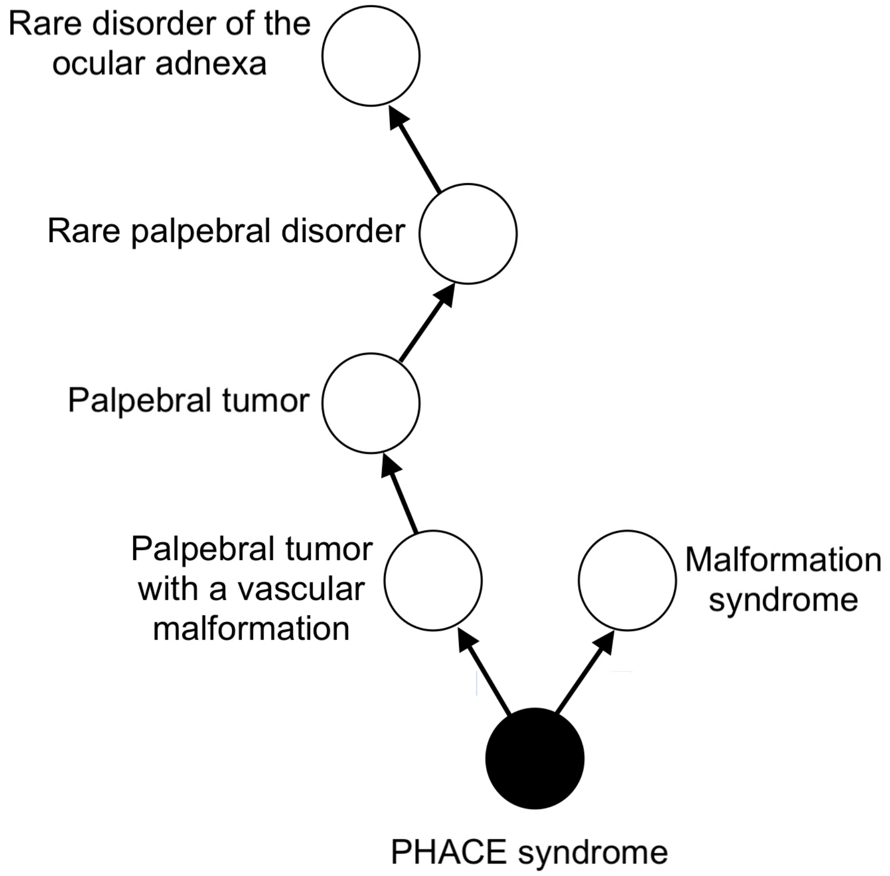

Table S1

	total terms at time of publication	terms reviewed during the ERN-EYE Ontology meeting, 10/2017
Human Phenotype Ontology (HPO), 11/2018 version	1106	605 added,<br>>400 revised
Orphanet Rare Disease Ontology (ORDO), 11/2018 version	1202	67 groups created,<br>36 disorders introduced,<br>90 terms removed/merged,<br>131 nomenclature modifications

A detailed list of the modifications that were made in HPO as a result of the ERN-EYE Ontology meeting can be found in the HPO GitHub repository (label "ERN-EYE": https://github.com/obophenotype/human-phenotype-ontology/labels/ERN-EYE). For a list of the groups and disorders introduced to ORDO please see Table S2 and https://www.orpha.net.

Table S2

A list of disease groups and specific disorders introduced to the Orphanet Rare Disease Ontology (ORDO) as a result of the ERN-EYE Ontology meeting (Mont Sainte-Odile, France, 10/2017)

ORDO disease groups	ORPHA code
Rare disorder of the visual organs	ORPHA:520814
Rare disorder of the orbital region	currently evaluated
Rare disorder of the ocular adnexa	ORPHA:519266
Rare eyelid disorder	currently evaluated
Rare nasolacrimal system disorder	currently evaluated
Syndromic nasolacrimal system disorder	ORPHA:519274
Isolated nasolacrimal system disorder	currently evaluated
Aplasia/hypoplasia of the nasolacrimal drainage system	currently evaluated
Inflammatory/autoimmune disorder involving the nasolacrimal system	ORPHA:519264
Rare disorder with entropion	ORPHA:519270
Rare disorder with ectropion	ORPHA:519268
Structural developmental eye defect	ORPHA:519272
Coloboma with extraocular manifestations	currently evaluated
Rare disorder of the anterior segment of the eye	ORPHA:519284
Anterior segment dysgenesis with extraocular manifestations	ORPHA:519276
Rare disorder with conjunctival involvement as a major feature	ORPHA:519280
Rare disorder with corneal involvement as a major feature	ORPHA:519288
Rare corneal disorder	ORPHA:519282
Rare inflammatory/autoimmune corneal disorder	ORPHA:519290
Non-infective crystalline keratopathy	currently evaluated
Infective keratitis	ORPHA:519278
Rare disorder of the pupil	ORPHA:519286
Early-onset cataract	currently evaluated

ORDO disease groups (continued)	ORPHA code
Syndromic ectopia lentis	ORPHA:519292
Syndromic microspherophakia	ORPHA:519294
Syndromic microphakia	ORPHA:519294
Rare scleral disorder	ORPHA:519298
Rare disorder with pigmented sclera	ORPHA:519296
Rare disorder of the posterior segment of the eye	ORPHA:519311
Rare choroidal disorder	ORPHA:519309
Isolated chorioretinal dystrophy	ORPHA:519300
Syndromic chorioretinal dystrophy	ORPHA:519321
Rare retinal disorder	ORPHA:519315
Inflammatory/autoimmune retinopathy	currently evaluated
Retinal tumor	currently evaluated
Rare retinal vasculopathy	ORPHA:519317
Isolated vitreoretinopathy	ORPHA:519304
Syndromic vitreoretinopathy	ORPHA:519327
Isolated inherited retinal disorder	currently evaluated
Isolated stationary inherited retinal disorder	ORPHA:519319
Isolated progressive inherited retinal disorder	ORPHA:519306
Syndromic inherited retinal disorder	ORPHA:519325
Syndromic stationary inherited retinal disorder	currently evaluated
Syndromic progressive inherited retinal disorder	currently evaluated
Inherited disorder of cone function	currently evaluated
Rare macular disorder	ORPHA:519313
Isolated macular dystrophy	ORPHA:519302
Syndromic macular dystrophy	ORPHA:519323
Rare disorder affecting multiple structures of the eye	ORPHA:519329
Primary early-onset glaucoma	currently evaluated
Secondary early-onset glaucoma	ORPHA:519331
Rare optic nerve disorder	ORPHA:519351
Rare disorder with optic disc malformation	ORPHA:519345
Rare ophthalmic disorder with cranial nerve involvement	ORPHA:519349
Rare oculomotor nerve disorder	currently evaluated
Rare trochlear nerve disorder	ORPHA:519353
Rare trigeminal nerve disorder	currently evaluated
Rare abducens nerve disorder	currently evaluated
Rare facial nerve disorder	currently evaluated
Rare disorder with extraocular muscle involvement	ORPHA:519355
Rare ocular motility/alignment disorder	ORPHA:519347
Inflammatory/autoimmune optic neuropathy	ORPHA:499047
Pseudopapilledema	ORPHA:519339
Disorder with optic nerve compression	ORPHA:519337
Congenital optic disc excavation	ORPHA:519333
Rare brainstem/cerebellar disorder with ophthalmic involvement as a major feature	ORPHA:519341
Rare cortical disorder with ophthalmic involvement as a major feature	ORPHA:519343

ORDO disorders	ORPHA code
Congenital cystic eye	ORPHA:519384
Isolated coloboma	currently evaluated
Isolated blepharochalasis	ORPHA:519390
Isolated congenital entropion	ORPHA:519386
Complete absence of nasolacrimal drainage system	currently evaluated
Agenesis lacrimal ducts	currently evaluated
Congenital lacrimal punctal membrane	currently evaluated
Autosomal recessive anterior segment dysgenesis	currently evaluated
Posterior polymorphous corneal dystrophy type 1	currently evaluated
Posterior polymorphous corneal dystrophy type 2	currently evaluated
Posterior polymorphous corneal dystrophy type 3	currently evaluated
Early-onset Fuchs endothelial corneal dystrophy	currently evaluated
Thygeson superficial punctate keratopathy	ORPHA:519406
Fungal keratitis	ORPHA:519930
Mooren ulcer	ORPHA:519408
Terrien marginal degeneration	ORPHA:519410
Infective crystalline keratopathy	currently evaluated
Paraproteinemic keratopathy	currently evaluated
Isolated iridoschisis	ORPHA:519392
Isolated microphakia	ORPHA:519394
Isolated microspherophakia	ORPHA:519396
Early childhood onset retinal dystrophy	currently evaluated
Isolated foveal hypoplasia	ORPHA:519398
Isolated megalopapilla	ORPHA:519402
Isolated morning glory anomaly	currently evaluated
Peripapillary staphyloma	ORPHA:519400
Optic disc pit	ORPHA:519404
Isolated optic neuritis	ORPHA:499096
Chronic relapsing inflammatory optic neuropathy	ORPHA:499085
MOG-positive optic neuritis	currently evaluated
GQ1b-positive optic neuritis	currently evaluated
AQP4-positive optic neuritis	currently evaluated
Optic perineuritis	ORPHA:499107
Osteopetrosis with optic nerve compression	currently evaluated
Ischemic optic neuropathy associated with optic disc drusen	currently evaluated
Ocular myasthenia	currently evaluated

“Currently evaluated” is used for terms that are under consideration as of 19 Nov 2018.
